# Prior Vaccination and Effectiveness of Communication Strategies Used to Describe Infectious Diseases

**DOI:** 10.3201/eid2504.171408

**Published:** 2019-04

**Authors:** Thomas S. Valley, Aaron M. Scherer, Megan Knaus, Brian J. Zikmund-Fisher, Enny Das, Angela Fagerlin

**Affiliations:** University of Michigan, Ann Arbor, Michigan, USA (T.S. Valley, A.M. Scherer, M. Knaus, B.J. Zikmund-Fisher);; University of Iowa, Iowa City, Iowa, USA (A.M. Scherer);; Radboud University Nijmegen, Nijmegen, the Netherlands (E. Das);; University of Utah School of Medicine, Salt Lake City, Utah, USA (A. Fagerlin);; Informatics Decision-Enhancement and Analytic Sciences (IDEAS 2.0) Center for Innovation, Salt Lake City (A. Fagerlin)

**Keywords:** communication, influenza, human, immunization, vaccination, viruses, vaccines

## Abstract

We tested the effect of prior vaccination on response to communication strategies in a hypothetical news article about an influenza pandemic. Vaccinated were more likely than nonvaccinated participants to plan future vaccination, and future vaccination intent was greater with certain communication strategies. Using these findings to target communication may increase vaccination rates.

Vaccination rates for influenza remain surprisingly low (*1*). Despite goals to vaccinate 75% of high-risk Europeans by 2010, <50% had been vaccinated in 2013 (*2*). The reluctance of at-risk persons to receive vaccinations highlights the challenge of broadly vaccinating the general public.

Improving communication strategies that clinicians and healthcare organizations use to increase vaccination rates is cost-effective (*3*). Yet randomized trials to improve influenza vaccination rates by improving physicians’ communication skills (*4*) or by using various public health messages (*5*) have not succeeded. Several studies have examined the effect of various communication strategies to improve vaccination rates for influenza (*6*–*9*). However, the greatest predictor of future vaccination is prior vaccination, and these studies assessed participants in aggregate (*6*). Guided by the Health Belief Model (*10*), we investigated whether experiences with prior vaccination might affect the effectiveness of certain communication strategies (Appendix).

Our study is a secondary analysis of a randomized experiment to test communication strategies and their effects on influenza immunization (*6*–*9*). After our study was deemed exempt from review by the University of Michigan Institutional Review Board, we recruited a stratified random sample of adults from a panel of Internet users through Survey Sampling International (https://www.surveysampling.com) (Appendix). We recruited participants from 11 countries: Finland (n = 1,554), Norway (n = 764), Sweden (n = 1,539), Hungary (n = 998), Poland (n = 1,509), Spain (n = 1,604), Italy (n = 1,509), Germany (n = 1,546), the Netherlands (n = 1,938), the United Kingdom (n = 1,762), and the United States (n = 1,787).

Participants read a hypothetical news article that described the spread of influenza in their country. The article directly quoted hypothetical health experts and contained information about the influenza virus, its potential symptoms, and a vaccine in development. Articles were cross-randomized to provide participants with 5 varying communication strategies: 1) graphics (heat map, DOT map, picto-trendline) (*6*); 2) case severity (severe, typical, both) (*9*); confident language (scientific certainty, uncertainty, uncertainty with normalizing language) (*7*); 4) influenza label (H11N3 influenza, horse flu, Yarraman flu) (*8*); and 5) metaphor use (infectious disease, war, gardening). The Appendix contains more information about communication strategies. Each news article contained all 5 communication strategies. The experiment used a 3 × 3 × 3 × 3 × 3 between-subjects factorial design in which participants were randomly assigned to each communication strategy. After reading the newspaper article, participants were asked their vaccination status (whether they had received an influenza vaccination within the past 2 years) and intent to get vaccinated in the future (defined by a discrete visual analog scale ranging from 1 [“Definitely would not get a vaccination”] to 7 [“Definitely would get a vaccination”]).

We were interested in the main effect for an individual communication strategy depending on a participant’s prior vaccination status. For each communication strategy, we conducted separate ordinal logistic regression models and included an interaction term of prior vaccination and the communication strategy of interest for each model. The dependent variable was intent to get vaccinated. As covariates, we included the participant’s age, sex, and marital status and whether the participant was a healthcare worker. We estimated robust SEs with clustering by the participant’s country of residence.

Of 20,138 participants, 16,401 (81%) completed the survey; of these, 4,999 (30%) had received an influenza vaccination within the previous 2 years and 11,402 (70%) had not. The average age was 51.4 (SD ± 16.9) for vaccinated and 44.9 (SD ± 15.4) for nonvaccinated participants. Approximately 44.6% of vaccinated and 52.1% of nonvaccinated participants were female (Appendix Table 1).

Our results showed that previously vaccinated participants were more likely than nonvaccinated participants to plan for future vaccinations (adjusted odds ratio 5.8, 95% CI 4.8–7.0; p<0.001). We found significant interaction effects between prior vaccination and each communication strategy (p<0.001 for each strategy) (Table; Appendix Table 2). However, this effect varied according to the type of communication strategy. Nonvaccinated participants reported greater intent for future vaccination when heat maps, severe cases, confident language, or exotic influenza labels were used (Table). Vaccinated participants reported greater intent for future vaccination when confident language or scientific/exotic influenza labels were used (Table). The use of metaphors had no effect on either group.

**Table:** Effect of communication strategies on intent for future influenza vaccination, by influenza vaccination status*

Strategy	Vaccination over previous 2 y, adjusted odds ratio (95% CI)*				p value for interaction†
No	p value	Yes	p value
Graph type					<0.001
Picto-trendline	Referent		Referent		
DOT map	1.1 (0.9–1.2)	0.06	1.0 (0.9–1.1)	0.92	
Heat map	1.1 (1.0–1.2)	0.01	1.1 (0.9–1.2)	0.08	
Case severity					<0.001
Both	Referent		Referent		
Typical	1.0 (0.9–1.1)	0.78	0.9 (0.8–1.0)	0.07	
Severe	1.1 (1.0–1.3)	0.02	1.1 (0.9–1.2)	0.43	
Confident language					<0.001
Uncertainty with normalizing language	Referent		Referent		
Uncertainty	1.0 (0.9–1.1)	0.97	1.1 (0.9–1.2)	0.12	
Scientific certainty	1.2 (1.1–1.3)	<0.001	1.3 (1.1–1.4)	<0.001	
Influenza label					<0.001
Horse	Referent		Referent		
H11N3	1.0 (0.9–1.1)	0.62	1.4 (1.1–1.7)	0.001	
Yarraman	1.1 (1.0–1.2)	0.001	1.2 (1.1–1.4)	0.001	
Metaphor use					<0.001
Infectious disease	Referent		Referent		
War	1.0 (0.9–1.1)	0.78	1.0 (0.9–1.1)	0.60	
Gardening	1.0 (0.9–1.1)	0.75	1.0 (0.9–1.1)	0.41	

*Multivariable ordinal logistic regression adjusted for participant age, sex, marital status, occupation as healthcare worker, and country of residence. †Interaction between vaccination status and communication strategy.

This study should be interpreted in the context of certain limitations. For instance, participants reviewed a hypothetical news article, which may be different than direct communication with a healthcare provider or reading an actual article during a pandemic.

Certain communication strategies, such as use of confident language or an exotic influenza label, were effective regardless of prior vaccination status. Yet use of a scientific influenza label was more effective than use of an exotic influenza label among previously vaccinated participants. Other communication strategies, such as use of heat maps or describing severe cases, were effective among nonvaccinated but not previously vaccinated participants. Vaccination rates for influenza may be improved by targeting healthcare communication based on prior vaccination experiences (*11*,*12*).

Appendix Additional methods and results for study of prior vaccination and effectiveness of communication strategies used to describe infectious diseases.
