# Supplementary material for: Prior Vaccination and Effectiveness of Communication Strategies Used to Describe Infectious Diseases
Source: Emerg Infect Dis. 2019 Apr;25(4):821–3. doi: 10.3201/eid2504.171408 (PMC6433032; doi:10.3201/eid2504.171408)
Supplement: Appendix — Additional methods and results for study of prior vaccination and effectiveness of communication strategies used to describe infectious diseases. [file 17-1408-Techapp-s1.pdf]

Article DOI: https://doi.org/10.3201/eid2504.171408

Prior Vaccination and Effectiveness of Communication Strategies Used to Describe Infectious Diseases

Appendix

Table of contents:

1. Appendix Methods

2. Example of a scenario provided to UK participants

3. Appendix Figure: Example of heat map provided to UK participants

4. Appendix Table 1: Respondent characteristics by vaccination status

5. Appendix Table 2: Effect of communication strategies on intent to vaccinate with no prior vaccination as the reference category

6. Appendix References

Methods

Theoretical frameworks

Prior studies have evaluated the effect of certain communication strategies to improve influenza vaccination rates (*1–4*). Yet, these studies looked at aggregate results among all participants. We hypothesized that people respond differently to messaging, particularly based on their belief system.

Thus, we used the Health Belief Model (HBM) as a theoretical framework for the hypothesis that guided this study (*5*). HBM was developed as an overarching concept that health behavior is based on personal beliefs about a disease (perceived benefits, perceived barriers, perceived susceptibility, perceived severity), available strategies to mitigate the disease (cues to action, self-efficacy), and modifying variables. We believed that prior vaccination might act on

several layers of the HBM—on perceived benefits, barriers, susceptibility, and severity. In other words, whether or not an individual receives a prior vaccination may be due to their health beliefs, and these health beliefs may affect how they respond to healthcare communication aimed at improving vaccination rates.

Study population

A stratified random sample of adults was recruited by Survey Sampling International (SSI) from a panel of Internet users. Panel members were recruited through various opt-in methods, such as Web sites, Internet banners, e-mails, television advertisements, e-mails, apps, and social media. SSI employs a probability-weighted random process to select panel members.

For this study, quotas were established based on age and gender to ensure that the sample was representative of these characteristics for each country. The sampling algorithm continued to recruit SSI participants until all quotas were achieved. Participants were recruited between February and March 2016. Incomplete surveys were excluded. Upon survey completion, participants were entered into drawings administered by SSI for modest prizes.

Subjects were recruited from Finland, Germany, Hungary, Italy, the Netherlands, Norway, Poland, Spain, Sweden, the United Kingdom, and the United States. Participants received surveys in the primary language of their country of residence. Countries were grouped into six regions for analyses. The regions were defined geographically: North (Finland, Norway, Sweden), East (Hungary, Poland), South (Spain, Italy), West (Germany, the Netherlands), the United Kingdom, and the United States.

Survey

Participants were requested to imagine an epidemic of influenza and then provided with a simulated news article that described the spread of influenza in their country. The article contained direct quotes from hypothetical healthcare experts, as well as information regarding the influenza virus, its potential symptoms, and a vaccine under development. Articles and surveys were translated from English to the country's main language and reviewed with a native speaker from each country.

Communication strategies

Five communication strategies were tested: graphics (*1*), case severity (*4*), confident language, influenza labels (*3*), and metaphor use.

For graphics, articles contained one of three visualizations presenting influenza prevalence (heat map, dot map, or picto-trendline) (*1*).

For case severity, the average case of influenza was either 1) not discussed, 2) described as mild (moderate fever and cough that is self-limited), or 3) described as severe (high fever, cough, vomiting that generally requires intravenous medication and hospitalization) (*4*).

For confident language, the article contained quotes from a hypothetical scientific expert, who used language of *scientific certainty* ("Health officials are confident that this outbreak will be a bad one."), *uncertainty* ("Yet, health officials say it's still too soon to tell just how bad the outbreak will be."), or *uncertainty with normalizing language* ("It's simply too early to predict how severe the flu will be. It might turn out to be mild to moderate like most seasonal flus but could also be more severe than usual.") (*2*).

For influenza labels, each article referred to influenza by one of three labels: 1) "H11N3 influenza," a scientific label; 2) "horse flu," an animal reservoir label; or 3) "Yarraman flu," an exotic-sounding label. Yarraman is an aboriginal term for horse (*3*).

For metaphor use, articles used one of three metaphor styles to describe the spread of the influenza pandemic: 1) infectious disease (using words such as virus and infecting); 2) war (using words such as invading, acts like an army, infiltrate, combat); or 3) gardening (springing up across, grown, acts like a weed) (*3*).

Data quality

All Survey Sampling International (SSI) participants undergo systematic quality controls before inclusion in any sample. For instance, SSI uses digital fingerprinting to flag duplicate respondents. SSI performs continuous monitoring to assess for inappropriately quick responses or inattention. To confirm location, SSI uses two-factor authentication before reward redemption.

Data analysis

Data management and analysis were performed using Stata 14.2 (StataCorp, College Station, TX). All tests were two-sided with P values less than 0.05 considered significant.

Example of a scenario provided to UK participants

Intro

Imagine there has been an outbreak of the flu. The following article that you will read describes the current status of the outbreak.

Scenario

PHE Reports H11N3 Influenza Spreading Across the UK

The H11N3 Influenza has been springing up across the United Kingdom. The number of people reported to have H11N3 Influenza has grown recently according to health officials at Public Health England (PHE).

Health officials are confident that this outbreak will be a bad one. “H11N3 Influenza acts like a weed quickly spreading across the UK,” says Dr. Peter Hamilton, the lead expert with the PHE. “We are seeing it spring up and move from city to city with alarming speed.”

“H11N3 Influenza is a severe virus, and people are at risk for serious illness or death,” said Dr. Hamilton. “Although we believe that many people will only have relatively mild to moderate symptoms, we expect to see some severe cases, some of which will lead to death.”

Most of those who have gotten sick have experienced moderate fever with cough and body aches. Symptoms generally go away without medicine. Some extreme cases have required patients seeing a doctor and 1–2 days of hospitalization. These individuals experienced difficulty breathing, sudden dizziness, and severe persistent coughing.

Dr. Hamilton emphasized that the estimates of the symptoms that those with H11N3 will experience are based on the information currently available to health officials.

With a growing number of cases of people getting the virus, Dr. Hamilton promised that the soon to be released vaccine will prevent people from getting H11N3 Influenza. Vaccines eradicate the spread of diseases by using the body’s natural response to prevent us from getting sick. Specifically, the H11N3 Influenza vaccine will create antibodies, which are like the gardeners of the body that identify weeds so the immune system can quickly uproot H11N3 Influenza when it is encountered again.

Dr. Hamilton assured that the vaccine will be safe, effective, and has been tested extensively. “The H11N3 Influenza vaccine uses many of the same elements of vaccines from previous flu seasons and is undergoing standard development and testing. We have every reason to believe the vaccine will be effective, and it’s the best option available right now to protect people against the H11N3 Influenza virus,” said Dr. Hamilton.

“The vaccine is the most effective way we have to prevent the growth of H11N3 Influenza,” he said. Once the vaccine becomes available, Dr. Hamilton urged people to get vaccinated, even if they have questions about their risks of H11N3 Influenza or the effectiveness of the vaccine.

References

1. Fagerlin A, Valley TS, Scherer AM, Knaus M, Das E, Zikmund-Fisher BJ. Communicating infectious disease prevalence through graphics: Results from an international survey. Vaccine. 2017;35:4041–7. PubMed http://dx.doi.org/10.1016/j.vaccine.2017.05.048

2. Han PKJ, Zikmund-Fisher BJ, Duarte CW, Knaus M, Black A, Scherer AM, et al. Communication of Scientific Uncertainty about a Novel Pandemic Health Threat: Ambiguity Aversion and Its Mechanisms. J Health Commun. 2018;23:435–44. PubMed http://dx.doi.org/10.1080/10810730.2018.1461961

3. Scherer AM, Knaus M, Zikmund-Fisher BJ, Das E, Fagerlin A. Effects of Influenza Strain Label on Worry and Behavioral Intentions. Emerg Infect Dis. 2017;23:1425–6. PubMed http://dx.doi.org/10.3201/eid2308.170364

4. Zikmund-Fisher BJ, Scherer AM, Knaus M, Das E, Fagerlin A. Discussion of Average versus Extreme Case Severity in Pandemic Risk Communications. Emerg Infect Dis. 2017;23:706–8. PubMed http://dx.doi.org/10.3201/eid2304.161600

5. Champion V, Skinner C. The Health Belief Model. Health Behaviour and Health Education; Theory, Research, and Practice. San Francisco: Jossey-Bass; 2008.

Appendix Table 1. Respondent characteristics by vaccination status

	Received vaccination in past two years		
Characteristic	No	Yes	All respondents
Respondents	11,402 (69.5)	4,999 (30.5)	16,401
Age			
<35	3,354 (29.9)	1,062 (21.7)	4,416 (27.4)
35–50	3,193 (28.4)	1,021 (20.8)	4,214 (26.1)
50–59	1,948 (17.4)	674 (13.8)	2,622 (16.3)
60+	2,733 (24.3)	2,146 (43.8)	4,879 (30.3)
Gender			
Male	5,361 (47.3)	2,718 (54.6)	8,079 (49.4)
Female	5,928 (52.1)	2,217 (44.6)	8,145 (49.8)
Other	87 (0.8)	39 (0.8)	126 (0.8)
Married	6,600 (58.0)	3,350 (67.2)	9,950 (60.8)
Healthcare worker	768 (6.8)	708 (14.3)	1,476 (9.1)
Region			
North	2,726 (23.9)	1,104 (22.1)	3,830 (23.4)
East	2,027 (17.8)	462 (9.2)	2,489 (15.2)
South	2,315 (20.3)	780 (15.6)	3,095 (18.9)
West	2,383 (20.9)	1,074 (21.5)	3,457 (21.1)
UK	1,080 (9.5)	907 (18.1)	1,752 (10.7)
U.S.	871 (7.6)	907 (18.1)	1,778 (10.8)

Appendix Table 2. Effect of communication strategies on intent to vaccinate with no prior vaccination as the reference category

	Vaccination over the past two years			
Strategy	aOR[a] for "No"*	*P* for "No"	aOR[b] for "Yes"†	*P* for row interaction‡
Graphic				
Picto-trendline	Reference	Ref	6.1 (5.0, 7.5)	<0.001
Dot map	1.1 (0.9, 1.2)	0.06	6.2 (5.0, 7.6)	<0.001
Heat map	1.1 (1.0, 1.2)	0.01	6.7 (5.7, 7.9)	<0.001
Case severity				
Both	Reference	Ref	6.4 (5.4, 7.6)	<0.001
Typical	1.0 (0.9, 1.1)	0.78	5.6 (4.6, 6.9)	<0.001
Severe	1.1 (1.0, 1.3)	0.02	6.8 (5.7, 8.1)	<0.001
Confident language				
Uncertainty with normalizing language	Reference	Ref	5.7 (4.7, 7.0)	<0.001
Uncertainty	1.0 (0.9, 1.1)	0.97	6.2 (5.2, 7.3)	<0.001
Scientific certainty	1.2 (1.1, 1.3)	<0.001	7.1 (5.9, 8.6)	<0.001
Influenza label				
Horse flu	Reference	Ref	4.7 (4.1, 5.4)	<0.001
H11N3	1.0 (0.9, 1.1)	0.62	6.5 (5.0, 8.3)	<0.001
Yarraman flu	1.1 (1.0, 1.2)	0.001	5.8 (4.9, 6.9)	<0.001
Metaphor use				
Infectious disease	Reference	Ref	5.9 (4.8, 7.3)	<0.001
War	1.0 (0.9, 1.1)	0.78	5.7 (5.0, 6.5)	<0.001
Gardening	1.0 (0.9, 1.1)	0.75	6.1 (5.2, 7.3)	<0.001

*Adjusted odds ratio (95% confidence interval). Multivariable ordinal logistic regression adjusted for participant age, gender, marital status, occupation as healthcare worker, and country of residence.
†Reference value for this column is the "No" column.
‡Test of row interactions between vaccination status and the communication strategy.

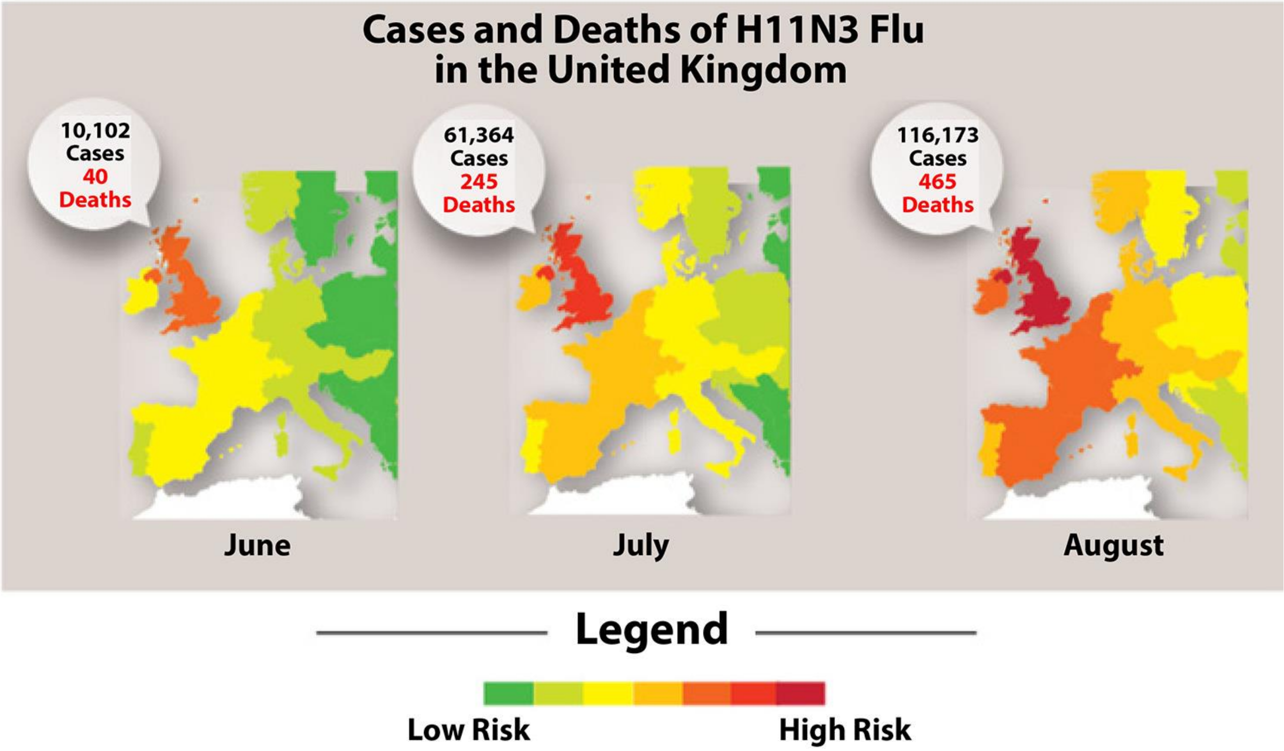

Appendix Figure. Example of heat map provided to UK participant.
